# Detection of circulating tumor cells: opportunities and challenges

**DOI:** 10.1186/s40364-022-00403-2

**Published:** 2022-08-13

**Authors:** Siwei Ju, Cong Chen, Jiahang Zhang, Lin Xu, Xun Zhang, Zhaoqing Li, Yongxia Chen, Jichun Zhou, Feiyang Ji, Linbo Wang

**Affiliations:** 1 Department of Surgical Oncology, The Sir Run Run Shaw Hospital Affiliated Hospital, Zhejiang University School of Medicine, Zhejiang, Hangzhou China; 2 Key Laboratory of Cancer Prevention and Intervention, Ministry of Education, Zhejiang, Hangzhou China

**Keywords:** Circulating tumor cells (CTCs), Liquid biopsy, Epithelial-mesenchymal transition (EMT), Isolation technologies, Precision medicine

## Abstract

Circulating tumor cells (CTCs) are cells that shed from a primary tumor and travel through the bloodstream. Studying the functional and molecular characteristics of CTCs may provide in-depth knowledge regarding highly lethal tumor diseases. Researchers are working to design devices and develop analytical methods that can capture and detect CTCs in whole blood from cancer patients with improved sensitivity and specificity. Techniques using whole blood samples utilize physical prosperity, immunoaffinity or a combination of the above methods and positive and negative enrichment during separation. Further analysis of CTCs is helpful in cancer monitoring, efficacy evaluation and designing of targeted cancer treatment methods. Although many advances have been achieved in the detection and molecular characterization of CTCs, several challenges still exist that limit the current use of this burgeoning diagnostic approach. In this review, a brief summary of the biological characterization of CTCs is presented. We focus on the current existing CTC detection methods and the potential clinical implications and challenges of CTCs. We also put forward our own views regarding the future development direction of CTCs.

## Introduction

Cancer metastasis is the primary cause of death worldwide and remains one of the prevailing challenges in curing cancer [1]. Most patients with metastatic disease receive systemic drugs to prolong survival and improve symptoms, but there is usually no cure and patients cannot achieve long-term survival. Metastasis is a multistep process involving intravasation, extravasation, migration and regeneration, in which cancer cells from a primary tumor detach and invade distant tissues using the bloodstream as a transport system [2, 3]. Cells that are separated from the primary tumor and travel through the bloodstream are called circulating tumor cells (CTCs) [4]. Understanding their part in the metastasis may contribute to better therapeutic management. In addition, CTCs can be extracted to detect the biological characteristics and molecular type of primary tumor cells. CTCs were reported for the first time as the presence of cells in blood that had the same size, shape, and appearance as those in primary tumors 150 years ago in 1869 by Ashworth Thomas Ramsden [5]. Since then, many studies have focused on exploring and developing efficient detection techniques as CTCs are noninvasive and accessible and could overcome the problem of tumor heterogeneity [5–7]. Significant leaps in the detection and characterization of CTCs have been achieved over the past two decades with new methods and devices emerging for CTC analysis. However, several challenges are associated with the isolation, detachment and detection of CTCs. Firstly, CTCs are infrequent and rare. Approximately 1–100 cells along with 10^6^–10^8^ red blood cells can be found per milliliter of blood [8, 9]. Secondly, as cancer cells are heterogeneous, a variety of groups of CTCs have significant variations in the expression of surface biomarkers [10–12]. Therefore, it is not easy to recognize different types of CTCs by the identical standard [13]. Finally, the nondestructive release of CTCs after the cells are captured on the surface effectively poses a challenge [14]. Herein, we systematically review CTCs, briefly provide an overview of their biology, and mainly investigate the current and emerging CTC detection techniques. Moreover, the clinical aspects of CTCs are described, and examples of how CTCs can participate in monitoring cancer development and drug therapy responses are discussed. Although the detection of CTCs is a promising technique for precision medicine, notably, there are still many unsolved problems. In this review, we present the existing challenges and offer our own insights into the future development of CTCs.

### Biology of CTCs

CTCs are considered to detach themselves from a primary tumor and pass through the bloodstream which can reflect metastasis, and several studies have shown their diagnostic and prognostic significance [5, 15, 16]. Through some newly developed high-throughput technologies, we can isolate these cells from the blood and conduct research at the single-cell level [14]. Over the course of disease or treatment, CTCs can provide a precise, dynamic, and treatment-related method to treat cancer.

### Steps of metastasis

**Fig. 1:**
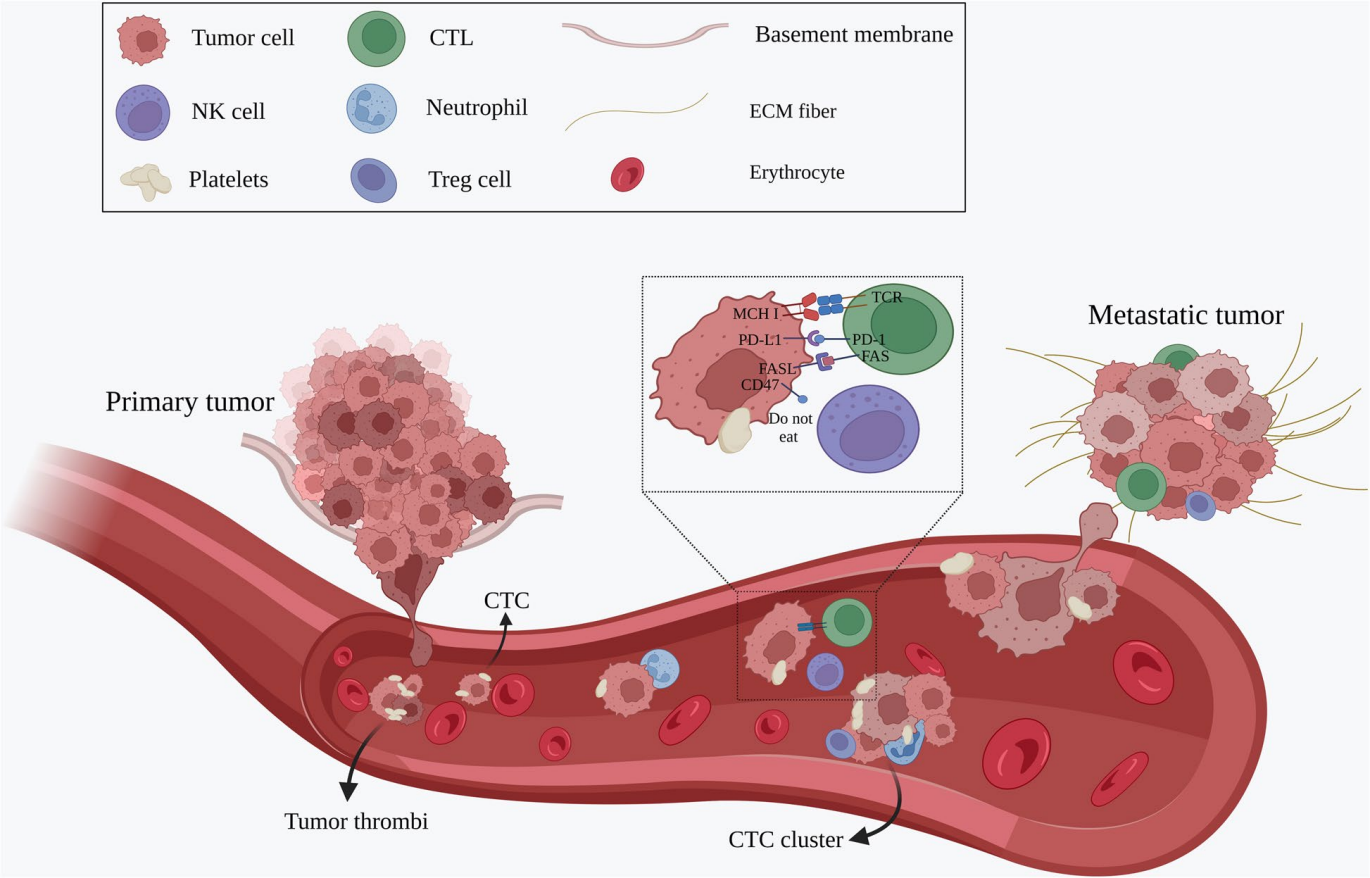
Tumor cell dissemination. 1) localized invasion; 2) intravasation; 3) survival in circulation; 4) arrest in the capillary bed; 5) extravasation and migration; 6) stimulation of angiogenesis. In addition to individual CTCs, CTC clusters are also found in patient blood, which have a significantly higher metastatic potential and increased ability to survive. CTCs get help from the platelets as well as immune cells during the escape phase

Tumor cell dissemination may occur in the following outlined steps: 1) localized invasion through the basement membrane during malignant progression [17]; 2) intravasation into hematogenous or lymphatic circulation systems, which allows for transport via circulation and interactions with blood components [18]; 3) survival in circulation by competition with circulating immune cells, loss of cell–cell junctions and shear stress [19, 20]; 4) arrest in the capillary bed of various organs [3]; 5) extravasation and migration into a foreign microenvironment, followed by colonization to form micrometastases [21]; and 6) stimulation of angiogenesis leading to growth into metastatic tumors (Fig. 1). However, this process is highly inefficient, and less than 0.01% of CTCs metastasize [22].

Most CTCs introduced into circulation are quickly killed by processes, such as immune attacks, shear stress, anoikis, oxidative stress and the lack of cytokines and growth factors [22]. Therefore, CTCs undergo a series of adaptations in order to survive in such a hostile environment. The epithelial to mesenchymal transition (EMT) has been identified as a vital process allowing CTCs to behave similar to mesenchymal cells [23]. During the EMT, epithelial cells lose epithelial characteristics such as the expression of EpCAM, keratins, and E-cadherin and upregulate matrix metalloproteinase (MMP) activity, which enables these cells to navigate through the local extracellular matrix (ECM) and enter the microvasculature [10, 24–26]. Thus, CTCs can be easily separated from a primary tissue, invade the capillaries and possess a significantly improved ability to survive and metastasize. In addition to individual CTCs, CTC clusters are also found in patients’ blood. CTC clusters are composed of 2 to 50 cells, including fibroblasts, endothelial cells, leukocytes and platelets, which have a prominently higher metastatic capacity and increased ability to survive [27].

### Characterization of CTCs

The isolation of viable CTCs enables the analysis of their molecular and functional characterization as CTCs are biochemically different from blood cells. One of the most common surface molecules on CTCs is the epithelial cell adhesion molecule (EpCAM), which originates from the epithelium [10, 25, 28]. EpCAM is a transmembrane glycoprotein that is present in 80% of solid cancers (such as breast, colorectal, and prostate cancer), but is absent in peripheral blood cells [10, 29]. Alternatives such as keratin 19, tumor-specific antigen 9, and progastrin-releasing peptides have also been reported [15]. Similarly, many tumor immune markers, such as prostate specific antigen (PSA), human epidermal growth factor receptor 2 (HER2) and endothelial growth factor receptor (EGFR), can also be used as antibodies for the specific recognition of CTCs [30, 31].

Nevertheless, in recent years, many studies have found that EpCAM is heterogeneously expressed or even not expressed on some cancers and cancer subtypes [32]. The process of the EMT has been found to be the most critical modulator of EpCAM expression [10]. The EMT is a strictly controlled process that allows cells to switch phenotypes. The EMT is believed to be essential for metastasis by promoting invasion, motility and dissemination in epithelial cancer cells [33]. Gorges and colleagues observed that EpCAM-negative breast cancer cells express high amounts of EMT-related genes [34]. The interrelated molecular mechanisms underlying EpCAM withdrawal from the cell surface may be associated with the endocytosis and subsequent degradation of EpCAM in intracellular compartments [25, 28, 32].

In addition to biochemical differences, there are distinct physical differences between CTCs and blood cells [35]. It is generally agreed that cell lines originating from solid tumors have greater cell sizes than blood cells. The cross-sectional areas of five tumor cell lines (MCF-7, Hep3B, HepG2, LNCaP, and HeLa) under a microscope were measured to be 396–796 μm^2^, which is significantly larger than that of leukocytes (average 140 μm^2^) measured under the same conditions [36]. Cell size extracted from dielectrophoresis (DEP) data clearly demonstrated the size difference among leukocytes (6.2–9.4 μm), leukemia cells (8.9–15.3 μm) and solid tumor cell lines (11.7–23.8 μm) [37]. Various biomechanical tools have been exploited to measure the mechanical properties of living cells, indicating that tumor cells with greater metastatic potential are more susceptible to deformation [24]. S. E. Cross and colleagues applied AFM to measure the stiffness of live metastatic cancer cells obtained from pleural fluids from patients with lung, breast and pancreatic cancer. The cell stiffness of metastatic cancer cells is more than 70% softer than that of benign cells within the same pleural fluid samples (Young’s modulus 0.53 ± 0.10 kPa versus 1.97 ± 0.07 kPa) [38]. Moreover, cancer cells contain a variety of polarizable particles, including peptides, proteins and nucleic acids. Gascoyne’s group applied dielectrophoretic field-flow fractionation (DEP-FFF) to study the dielectric properties of cancer cells and reported that the capacitances of cancer cells are significantly larger than those of blood cells. All data show that the total cell capacitance scales with the cube of the cell diameter, which is consistent with the general conclusion that cancer cells are larger than blood cells [37].

### Cells contribute to the survival of CTCs

Circulating tumor cells receive help from other nontumor cells during the escape phase (Fig. 1). Morphologic observations of tumor cells arrested in capillaries have documented the close association of tumor cells with activated platelets [39]. Platelets can rapidly enfold CTCs, protecting them from fierce shear forces [20]. Platelet aggregation induced by tumor cells can promote extravasation and adhesion [40]. Platelets also provide a defense against the immune system. Platelet-secreted transforming growth factor-β(TGF-β) is able to inactivate natural killer (NK) cells [41]. Transferring the MHC I complex from granular platelets to CTCs shields CTCs from the cytotoxic attack of NK cells [42].

In addition to platelets, increasing evidence suggests that many other blood cells are associated with the metastasis of CTCs in the bloodstream, such as neutrophils, monocytes and Treg cells. CTCs interact with endothelial-bound neutrophils in the vascular network, promoting adhesion and migration activities through different molecular targets (IL-8, CAM-1) expands the metastatic potential [19, 43, 44].

It has also been demonstrated that monocytes may play an important role in metastasis. Monocytes were observed to be associated with five or more CTCs in metastatic breast cancer (MBC) [45]. Classical monocytes can extravasate and differentiate into macrophages, promoting tumor cell extravasation, survival, and subsequent growth [46]. A subpopulation of CCR2 (receptor for chemokine CCL2) expressing monocytes was recruited by metastatic tumor cells which enhanced the subsequent extravasation of the tumor cells through the targeted delivery of molecules such as vascular endothelial growth factor(VEGF) [47].

CTCs have also adapted to avoid attack by immune cells in the bloodstream. Tumor cells are able to achieve immune escape by upregulating the expression of FASL on their surface, reducing the threshold for apoptosis in cytotoxic T lymphocytes (CTLs) [48]. Moreover, CTCs express programmed cell death-ligand 1(PD-L1), representing a potential mechanisms responsible for immune escape [49, 50]. Researchers have proposed that CTCs positive for PD-L1 can mediate Treg cells to play a role of immunosuppression. Treg cells can protect CTCs against being attacked by the immune system, weaken CTL killing ability and trigger more myeloid-derived suppressor cells (MDSCs) [51]. It has also been found that CTCs of colorectal cancer exhibit a distinct nonimmunogenic phenotype by overexpressing CD47 [52].

### Techniques used in CTC

As a consequence of the low concentration of CTCs in peripheral blood (1-100 cells per ml), a high specificity and an excellent affinity are both obligatory requirements for effective CTC capture as stated above. Most of the extant technologies consist of a two-step process of cell enrichment and subsequent detection. Most CTCs enrichment methods utilize the unique surficial antigen expression of CTCs to separate them from the great number of leukocytes, erythrocytes and other blood components [4, 53]. There are also technologies capturing CTCs that utilize the physical properties of CTCs including their size, density, and capacitive character [6, 54, 55]. The subsequent challenge is to effectively release CTCs from surfaces using enrichment methods without damaging the target cells14. Enzymatic digestion, oligonucleotide-mediated aptamer release, and stimuli-responsive polymers hold marvelous potential for CTC detachment [56–58].

### CTC isolation

**Fig. 2:**
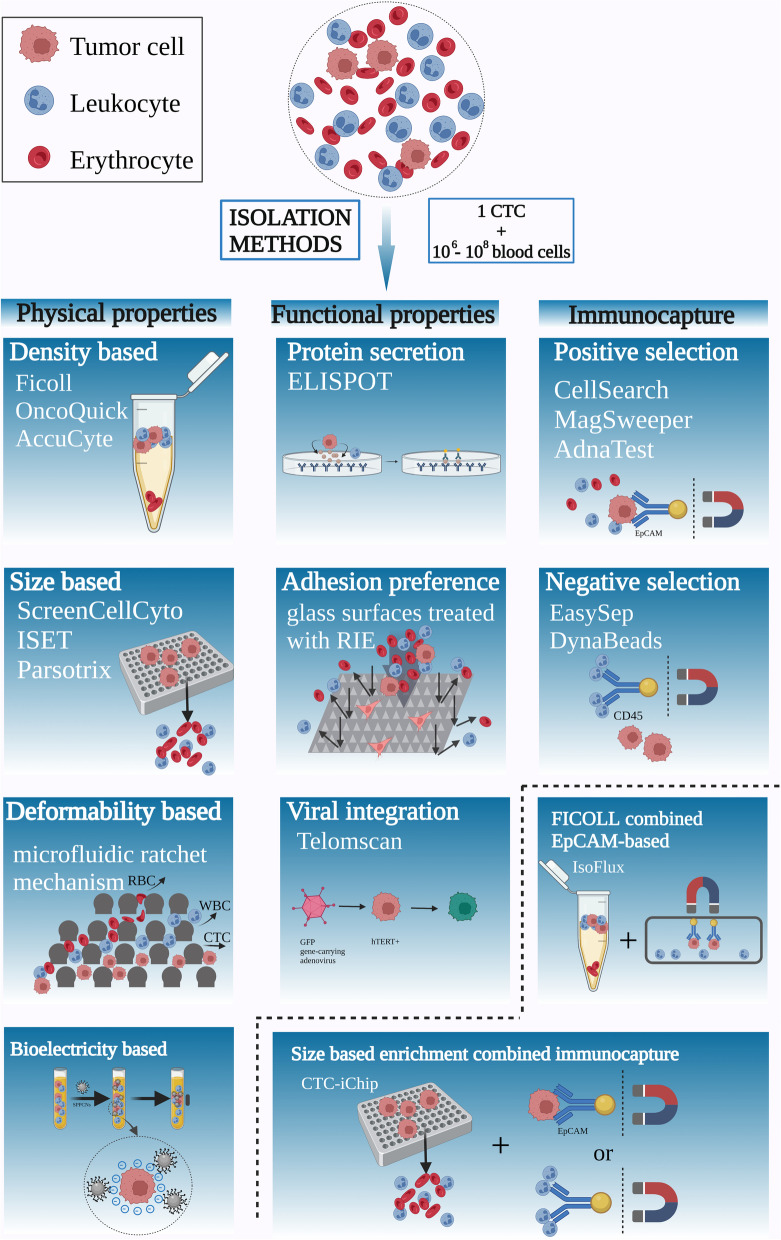
Outline of existing isolation techniques. The majority of CTC enrichment methods utilize the unique surficial antigen expression of CTCs or the physical or functional property of CTCs to separate CTCs from the great number of erythrocytes, leukocytes, and other blood. EpCAM: epithelial cell adhesion molecule; RBC: red blood cell; WBC: white blood cell; CTC: circulating tumor cell; GFP: green fluorescent protein; hTERT: human telomerase reverse transcriptase; SPPCNs: superparamagnetic positively charged nanoparticles

In view of the low abundance of CTCs in whole peripheral blood, separating CTCs from the background contamination of blood cells is a crucial step for subsequent analysis. However, the low frequency of CTCs along with the heterogeneity observed in CTCs render high-precision detection laborious. Currently, there is no ideal device capable of isolating a pure population of CTCs. Most separation methods are based on the physical properties or biological properties of CTCs. Due to limitations, such as low cell recovery, poor purity, and diminished viability, the widespread use of CTCs in laboratory and clinical environments is hindered (Fig. 2).

#### Devices utilizing physical properties

Enrichment methodologies via physical properties are based on unique CTC properties such as their size [50], membrane capacitance [6], and density [55]. The most essential advantage associated with the technologies above is that they are independent of the recognition of surface markers. Therefore, these techniques are appropriate for isolating CTCs with low/negative EpCAM expression levels as CellSearch® system fails to detect CTCs in approximately 36% of metastatic breast cancer and lung cancer patients [59, 60]. Isolation by the size of epithelial tumor cells (ISET®), Metacell filtration device, ScreenCellCyto, Parsotrix™, and dead flow fractionation techniques are all size-based CTC selections [50, 61–65], that utilize filtration to separate individual tumor cells on the basis of size. Coumans and colleagues compared three filtrations to investigate the properties of the ideal filter for CTC recovery, such as pore size, number of pores, spacing between pores, filter thickness and filter surface material. The authors summarized the experiment and arrived at the conclusion that the optimum filter for CTC enrichment from 10 ml of whole blood has a pore size of about 5 mm, a thickness of at least 10 mm, no less than 100,000 regularly spaced pores, and a porosity of 10% or less [66]. Unfortunately, these technologies have certain limitations, since the current technology lacks specificity, and due to the heterogeneity of cells, the results obtained are not as pure as those of functional tests. Meanwhile, filter pores can cause deformation and damage of CTCs and may lose CTCs with smaller sizes than average. On the other hand, larger cells that are not tumor cells, such as megakaryocytes, can be kept together with isolated cell populations. In conclusion, size-based CTC isolation methods provide high throughput; however, these methods have limited applicability in clinical settings due to the heterogeneity of CTCs in terms of their size.

Cell sorting based on deformability is particularly relevant to the separation of CTCs from whole blood because CTCs may not be simply distinguished from white blood cells based on size alone. This situation is particularly related to colorectal cancer and prostate cancer, as it is recognized that patients’ CTCs are small and have important overlap with contaminated leukocytes [67]. Tumor cells form an enlarged nucleus and, therefore, may exhibit a greater nucleocytoplasmic ratio than leukocytes. In fact, the nucleoplasm is two times more viscous and nearly three to four times more rigid than the cytoplasm. Thus, it seems to be a feasible option to take advantage of cell deformability to sort CTCs. Park and colleagues proved that separation based on deformability improves enrichment ≈100× over size-only separation, providing a significantly selective biophysical enrichment process. Their measurement involving the diameter of enriched CTCs and patients leukocytes before and after enrichment demonstrated that these cells were primarily discriminated on the basis of their cell deformability [67].

Centrifugation, which uses the specific density of leukocytes, red blood cells, and cancer cells, is among the first reported techniques used for CTC separation [68]. In density centrifugation methods, erythrocytes, platelets, and polymorphic nuclear cells are separated in the pellet, and mononuclear cells (MNCs), including tumor cells, gather in the so-called interphase [9]. A comparison of two density gradient centrifugation systems demonstrated that OncoQuick improved tumor cell enrichment in comparison with Ficoll, which was achieved by an increased consumption of MNCs and a comparable tumor cell recovery [55]. The AccuCyte® system is differentiated from existing density-based methods which separates the buffy coat from red blood cells and plasma by using a unique separation tube and collector device. The device allows virtually complete harvesting of the buffy coat into a small volume for application on a microscopic slide without cell lysis or wash steps, which is considered a potential source of CTC loss [69]. RosetteSep is an immune density cell separation kit designed to separate and enrich circulating epithelial tumor cells from normal hematopoietic cells. This kit contains an antibody cocktail for the removal of unwanted cells by changing their density. The excess cells settle through density gradient centrifugation, and purified tumor cells appear at the interface between the density gradient medium and the plasma [70].

Cancer cells have larger folding factors and radii than both normal cells of comparable origin and blood cells. The NCI-60 panel of cancer cell types has a DEP characterization and all cell lines derived from solid tumors have crossover frequencies that should allow their efficient isolation from normal blood cell types [6]. DEP is an electrokinetic method which allows inherent dielectric properties of suspended cells for discrimination and separation [71]. DEP has emerged as a promising method for isolating CTCs from whole blood. DEP isolation of CTCs is independent of cell surface markers [6]. The continuous flow ApoStream® device was developed to overcome the cell throughput limitation of the DEP batch mode configuration, providing an effective enrichment and separation of CTCs from full blood. The linearity data and recovery accuracy of the ApoStream® device confirmed consistent cancer cell recovery performance in both high- and low-EpCAM expressing cancer cell types over a wide range of spiking levels [72].

Bioelectricity is an essential biophysical indicator of cell behaviors and is directly modulated via the metabolic mode [73]. Successful CTC isolation based on the surface charge is uniquely related to the unique characteristics of cancer cells, i.e., a high glycolysis rate and strong lactate acid secretion. Studies have shown that the acidic cancer microenvironment associated with the “Warburg effect” is related to the negative charge of cancer cells, which is related to the following hallmark feature of cancer cell energy metabolism: a high aerobic glycolysis rate [74]. Superparamagnetic positively charged nanoparticles (SPPCNs) electrostatically and strongly bind malignant cells characterized by high rates of glycolysis, enabling the effective capture of CTCs and subsequent magnetic isolation from clinical blood samples of cancer patients [75].

#### Devices utilizing functional assays

Recent studies have reported that the EMT plays a pivotal role in the invasion of tumor cells [23, 76]. CTCs are hypothesized to contain a significant number of EMT tumor cells, which are reported to have a low expression of epithelial surface antigens, especially EpCAM [77]. Thus, such tumor cells that lose EpCAM expression are less likely to be detected by assays utilizing cell immunological characteristics. Functional assays use the viable CTC cellular functions to overcome certain limitations of tumor cell heterogeneity. However, the current circumscription of product purity is a dominant problem in the method of enriching CTCs based on cell functional characteristics. An adaptation of enzyme-linked immunospot technology, Epithelial Immunospot (ELISPOT), was introduced for the detection of viable CTCs in cancer patients. Secreted, shed or released proteins are immunocaptured on the membrane during short-term cultures, and the EPISPOT assay has a sensitivity to detect them [78]. In order to collect CTCs with aggressive phenotypes and explore their molecular features, researchers applied a functional cell separation method called the collagen adhesion matrix (CAM) assay, which identified CTC invasiveness via CAM protein uptake while recognizing epithelial antigens and produces results with high sensitivity and specificity [79].

Chen and colleagues reported a strategy for CTC enrichment by exploiting the differential adhesion preference of cancer cells to nanorough surfaces. Bare glass surfaces treated with reactive ion etching (RIE) for different durations could acquire different levels of roughness. Subsequently, RIE-generated nanorough surfaces could capture different types of cancer cells efficiently without any use of capture antibody [80]. This method is a promising strategy for achieving efficient capture at a quite low cost and is expected to provide a better isolation and enrichment strategy for viable CTCs from blood specimens. However, these nanorough glass surfaces show a low CTC capture purity as a result of significant nonspecific binding of other blood cells.

The telomere length has been frequently used as a means to predict the future life of cells [81]. TelomeScan detects viable CTCs via a telomerase-specific replication selective adenovirus in human peripheral blood. Viral infection increases the signal-to-background ratio as a tumor-specific probe and emits fluorescence. The transfected cells are easily recognizable, as the special adenovirus can be amplified only in tumor cells [82]. The TelomeScan may be more applicable for the detection of EMT tumor cells given that it is not influenced by the level of EpCAM expression. The assay seems promising, but future studies covering a large number of patients are still needed confirmation [83].

Tannic acid-functionalized magnetic nanoparticles (MNPs-TA) were recently developed for binding between the polyphenol structure of TA and the special glycocalyx on cancer cells. Furthermore, TA has a great antileukocyte adhesion effect, reducing the interaction with nontarget cells [84].

#### Devices utilizing Immunomagnetic assays

The most common technique used for CTC isolation is the immune isolation, which is based on specific CTC cell surface markers. Immunobead methods use either positive selection, targeting tumor-associated antigens expressed by CTCs, or negative selection, removing blood cells with common leukocyte biomarkers. EpCAM is an antigen often used in positive selection, while CD45 is used for negative selection [7, 85]. In 2004, the CellSearch® system was introduced as the first and only Food and Drug Administration (FDA)-approved method for the enumeration of CTCs in 7.5 mL of blood. The highest proportion of positive specimens was detected in patients with metastatic prostate cancer, followed by metastatic ovarian cancer and breast cancer [5]. The limitation of the fact that CellSearch® detects EpCAM+ cells leading to the loss of EpCAM-cells has been improved in other methods by combining different specific tumor markers, including EGFR, cytokeratin, HER2, folic acid receptors (FRs), and recombinant VAR2CSA (rVAR2) [16, 86–88]. In recent years, several alternative immunobead technologies that can improve the purity and recovery and retrieve CTCs off-chip with high fidelity have been developed, involving MagSweeper [53], AdnaTest® [43, 89], IsoFlux™ [90], and CTC-μChip [91].

Recently, researchers have combined CTC specific surface markers with other methods to improve the sensitivity of CTC detection and maintain the integrity and biological characteristics of CTCs for subsequent studies. Park and colleagues developed a novel 3D printed immunomagnetic concentrator (3DPIC) with an ATP luminescence assay for CTC enrichment and rapid detection. The ATP luminescence assay is used to measure cell intracellular ATP but has not been applied to CTC detection as a consequence of interference from non-CTC-derived ATP. An antibody (Ab)-conjugated magnetic nanoparticles (MNPs) conjugated with EpCAM provided spectacular enrichment in 3DPIC, and then, these cells were enumerated using an ATP luminescence assay [92]. Researchers have presented chemically stable and instantly degradable (CSID) hydrogel immunospheres for CTC isolation. These researchers modified the CSID hydrogel spheres with the anti-EpCAM antibody to successfully isolate and effortlessly retrieve the target cells with an average of 10.8 ± 5.9 CTCs/ml [93].

Negative selection is also a possible solution to overcoming the low recovery rate associated with EpCAM-cells. CD45 expressed in hematopoietic cells is the most prevailing antigen used in negative selection. CD45 depletion is often combined with other label-free methodologies, such as density gradient centrifugation or red blood cell lysis to improve the yield [85, 94]. Using these strategies, unconventional CD45-expressing CTCs may be accidentally removed, resulting in the underestimation of the number of CTCs [2]. DynaBeads® and EasySep are immunomagnetic methods in which antibodies recognizing cell surface antigens are coupled to magnetic beads and used to remove unwanted cells. However, it is not easy to enumerate massive Dynabead-bound cells due to the autofluorescence of the beads, the large number of beads, and the low efficiency of the labeling of cell antibodies when the beads are bound. In contrast, the smaller EasySep nanoparticles do not interfere with downstream immunocytochemical processing and are able to achieve higher purity [95].

#### Devices utilize microdevices

Nowadays microfluidic platforms have become among the most prevalent technologies because of their tremendous applications, including biological and chemical analyses, fertility analyses, cell sorting, infectious disease diagnostics, DNA sequencing, ect [96]. Microfluidic platforms provide many attractive advantages, such as continuous sample processing to reduce target cell loss. Microfluidic platforms can capture CTCs through different methods, which can be roughly divided into: 1) using epithelial cell markers as antigens; 2) using the physical properties of tumor cells; 3) using the electrical properties of CTCs; and 4) other methods [97].

A mass of microdevices utilize the unique antigen expression of CTCs as enrichment and capture methods. EpCAM, as already mentioned, is logically one of the most common surface markers applied to distinguish CTCs from hematopoietic cells [98–101]. An effective microfluidic named CTC-chip using antibody-coated microposts to capture these EpCAM-positive cells was demonstrated. Stott and colleagues developed a high-throughput microfluidic mixing device, the herringbone-chip (HB-Chip) which could provide an enhanced efficiency of CTC isolation. The HB-Chip design applies passive mixing of blood cells through the generation of microvortices to significantly increase the interactions between the target CTCs and the antibody-coated chip surface [98]. The electrochemical Lab-on-a-Disc (eLoaD) platform captures cancer cells from separated plasma through anti-EpCAM antibodies immobilized on gold electrodes and quantifies them by the use of label-free electrochemical impedance [99]. Lin and colleagues developed three generations of NanoVelcro CTC chips, a nanostructured substrate, coated with anti-EpCAM antibodies. These authors developed a third generation NanoVelcro device using biotin-streptavidin interaction linked anti-EpCAM antibodies to efficiently capture CTCs. At 37 °C, functionalized domains are present on the surface of the chips; thus, CTCs that interact with the substrate can be caught. The thermally responsive polymer brushes of a poly N-isopropylacrylamide (PIPAAm) substrate undergo conformational changes when the temperature decreases at 4 °C, leading to the internalization of the anti-EpCAM antibodies. Thus, the captured CTCs are capable of being released from the device [100]. A recent study presented a fully automated and rapid microfluidic system for efficient CTC identification. A lateral flow-based four-channel microfluidic chip was applied to separate and distribute CTCs as a single-cell array. An approximately 90% capture rate was achieved in different cell lines when spiking 100 cells in 2 mL of healthy donor blood samples from healthy donors, revealing the wide application of this platform to different tumors [102].

It has been proven that metastatic cancer cells from patients with lung, breast and pancreatic cancer are 70% softer than benign cells from the body cavity through atomic force microscopy (AFM) [52]. The iterative mechanical characteristics (iMECH) analyzer provides a low-cost yet high-throughput solution for single-cell level metastatic detection. It directs the cyclic deformation regimen by pulling CTCs and other cells through a trial channel composed of narrow deformation channels interspersed with wider relaxation regions to simulate the dynamic microenvironment jointly. Researchers revealed that cells from nonmetastatic breast cell lines were more resistant to deformation when passing through cyclic deformations, and their average velocity through the channels decreased after each relaxation [103].

Alternatively, inertial microfluidics devices isolating cells based on size by utilizing the fluidic forces in straight or curved channels have already been developed [104, 105]. These devices demonstrated remarkably higher flow rates than immunoaffinity based devices, allowing a high throughput process. Researchers devised an efficient inertial device, the CTCKey™ without additional preprocessing steps. The study reported that CTCKey™-enriched blood could be further processed utilizing the CellSearch® system, enabling processing higher volumes of blood (up to 5fold) [105].

Over the past decade microdevices have emerged as promising techniques to address challenges, given rarity, phenotypic and size heterogeneities of CTC, and the need to preserve CTC viability for downstream analysis. Nevertheless, there are still certain limitations of these novel technologies. Physical-based microdevices face with the risk of clogging, low purity, and challenging downstream analysis. For example, the throughput of microfluidic ratchets is relatively low with 1 mL/h. The device fails to process 7.5 mL of blood (standard volume for protocols), which may overstate the probability of recovering CTCs in the clinic trail with microfluidic ratchets [67]. The use of nanostructured substrates, such as silicon nanopillars (NanoVelcro Chip), was also reported to enhance CTC isolation sensitivity as the consequence of high surface area-to-volume ratio of nanostructured substrates and similar size to cellular surface components [90]. Moreover, subsequent enzymatic degradation may compromise the viability of CTCs due to over exposure to the degrading membrane itself and enzymatic hydrolysis solutions. The irreversible capture of CTCs on these nanostructures greatly limits downstream analysis and subsequent cell culture [56]. The immunomagnetic separation can either target CTCs or WBCs. As previously mentioned, such tumor–antigen dependent immunomagnetic separation methods are unable to overcome marker expression variability among CTCs. Nonetheless, given the high concentration of WBCs in blood, it is more challenging to deplete WBCs completely without CTC damage. In addition, these devices have to processed large volumes of blood to ensure the sufficient number of CTCs, which might lead to clogging on account of the large number of WBCs.

GILUPI GmbH CellCollector, an in vivo and novel technology, uses an anti-EpCAM wire directly into the peripheral arm vein and captures targeted cells with high efficiency. In the study, all volunteers tolerated the 30 min in vivo exposure to the nanodetectors with no sign of adverse events. Within the test 24 cancer patients were examined; of those, 22 of 24 were detected with a median of 5.5 (0–50) CTCs in breast cancer (*n* = 12) and 16 (2–515) CTCs in non-small cell lung cancer (NSCLC) (*n* = 12). This technology has the ability to process approximately 1.5 L of blood in 30 minutes, which improves the device’s sensitivity, thereby rendering it a promising candidate for future CTC studies [106].

Since the number of CTCs is very tiny and the time of tumor cells shedding into the blood might be related to biological rhythms. The fluorescence in vivo flow cytometry (IVFC) is developed as an emerging and powerful optical technique. The biggest advantage of IVFC is that the blood collection is not required. This technique could detect fluorescent circulating cells in living animals through a noninvasive manner over a long period of time to reduce the error caused by acquisition time [107]. This method helps to identify the effects of treatments, as sorafenib was revealed to reduce CTC count through fluorescence IVFC [108]. Nevertheless, IVFC has limitations: 1) its detection speed is 1 μL/min, while ~ 5 L/min blood passes through human blood vessels; 2) this emerging technology is still at the stage of animal models due to the use of fluorescent dyes; 3) it is unfavorable for the detection of CTC molecular typing and the study of biological characteristics. However, this technique is particularly useful for CTC detection and counting, which should be valuable for clinical monitoring and prognosis evaluation.

The advantages and limitations of various CTC isolation and detection technologies are summarized in Table 1.

**Table 1:** Summary of the CTC isolation and detection technologies

Name	Basics Properties	Limitations	(Pre)clinical Application	Recovery rate (%)	Reference
**Immunobead Assays**					
CellSearch® system	consists of ferrofluids coated with epithelial cell specific EpCAM antibodies; fluorescent labeling; immunohistochemical techniques	omitting CTCs expressing low levels of EpCAM; low frequency and large blood volumes demand; cells captured appear to be more apoptotic	FDA approved for advanced prostate, breast and colorectal cancers	≥85	[5, 7]
Weissenstein et al	a combination of anti-EpCAM and anti-cytokeratin magnetic cell separation	cells captured appear to be apoptotic	CTC levels were measured in MBC patients to assess prognostic value	78-90	[16]
MagSweeper	anti-EpCAM antibody-targeting immunomagnetic beads; characterize cells for multiple marker	omitting CTCs expressing low levels of EpCAM; cells captured appear to be apoptotic	capture live CTCs from BC patients for single cell analyses	55-69	[53]
IsoFlux™ Rare Cell Access System	a combination of flow control and immunomagnetic capture; Multiple kits for lab usage are available for cell enrichment and downstream analysis	Maximum daily analysis is 12 samples biomarker heterogeneity of CTCs	patients presenting ≥4 CTCs per blood draw were analyzed with prostate and colorectal cancers; KRAS mutations rate in CRC were described	≥74	[90]
AdnaTest®	incubate blood samples with an antibody mixture (e.g. anti-EpCAM and anti-MUC1); a magnetic particle concentrator extracts the labeled cell detect CTCs via RT-PCR assay for tumor-associated transcripts	biomarker heterogeneity of CTCs; magnetic beads may attach to the tube wall	a combined analysis of CellSearch® and AdnaTest® leads to an improved detection of CTCs in mCRC patient; prognosis prediction and efficacy evaluation in breast, prostate cancer	88	[43, 89, 109]
CTC-μChip	incubation with anti-EpCAM targeting immunomagnetic nanobeads; characterize gene expression using RT-ddPCR	omitting CTCs expressing low levels of EpCAM	CTC enumeration and genetic analysis in blood of patients with prostate cancer	> 90	[91, 110]
DynaBeads®	bind to desired target and beads responding to magnetic field	Only 3 types of DynaBeads are available for human tumor cell isolation	enumerate CD4+ T lymphocytes in HIV-1-infected individuals; not test in cancer patients	44 ± 23	[94]
MACS	CD45 leukocyte depletion method; utilize cytokeratin immunocytochemistry to analyze enriched cells	CTC expressing CD45 maybe remove from the sample; erythrocyte lysis cause damage to CTCs	CTC enumeration used in breast, lung, liver esophageal cancer patients; morphologically intact tumor cells were not detected in the clinical application	70-88	[111, 112]
EasySep	anti-CD45 for removal of blood leukocytes	CTC expressing CD45 maybe remove from the sample	CTCs were detected in all of the BC patients (23/23)	24 ± 19	[94, 113]
GILUPI CellCollector	ex vivo Functionalized Structured Medical Wire is antibody coated and applied into peripheral arm vein Isolation in vivo and overcomes sample blood volume limitations	Only used for extraction of CTCs directly from patient’s bloodstream	in vivo isolation of CTCs in patients with different stages of prostate cancer; distinguish between CTCs isolated from benign and malignant nodules	41	[106, 114, 115]
3DPIC	incubation with anti-EpCAM targeting immunomagnetic nanobeads Utilize ATP luminescence assay for the detection of cancer cells in blood	extracellular ATP derived from non-CTCs may interfere with the measurement	the ATP luminescence assay can detect as low as 10 cells in blood; not test in cancer patients	80	[92]
**Physical Property-Based Assay**					
microfluidic ratchet mechanism	distinguish CTCs based on cell deformability; deform cells in continuous flow without accumulating cells in the separation microstructure; the separated cells are available for downstream characterization	cellular damage; throughput limitation	detect CTC in a considerable proportion with clinically localized PC patients	> 90	[67, 116]
ISET®	Utilizes a filter-based, size exclusion approach to isolate epithelial cells; high throughput	morphology and size heterogeneity; damage or fragment CTCs on the result of multi-step cell processes	ISET® has a relatively good detection rate for CTCs in BC and NSCLC patients; fail to provide more information on pathological staging and molecular classification	75	[63, 64]
Metacell Filtration®	Size based separation technique driven by capillary-action; allow cytomorphological and immunocytochemical analysis of CTCs	Filters have a larger pore size (8 μm)	CTCs were detected in 66.7% evaluable PaC patients and the captured cancer cells displayed plasticity	66.7	[60, 117]
ScreenCell	size-based microfiltration; high CTC capture efficiency with processing 3 ml of blood per sample	unable to capture CTCs smaller than WBCs; erythrocyte lysis may cause damage to CTCs	the presence of CTCs does not influence prognosis in operated patients with NSCLC	89	[9, 118]
Parsotrix™	size and compressibility-based platform for CTCs isolation; ability to capture CTC clusters; harvests CTCs with both epithelial and mesenchymal features	CTC heterogeneity regarding size	Parsortix-enriched and stained cells were successfully transferred with preservation of cell morphology; not tested in clinical application	> 90	[65, 119]
Dielectrophoresis (DEP)	isolation based on polarizability and size; discriminate between cells of similar size having different morphological origins	requires specific parameters such as cell type and electric field frequency; the extent to which DEP will be applicable of CTC isolation in different types of cancer is unclear	concentrate MCF7 cancer cells from leukocytes; not test in cancer patients	> 90	[51, 120]
OncoQuick	polypropylene tube is inserted above the separation medium which allows for elimination of unwanted blood cells; High throughput, inexpensive	loss of sample while depleting mononuclear cells; detection depends upon only cytokeratine-20 biomarker	detect epithelial cells by RT-PCR targeting CEA, CK20, and TEM-8 in colorectal carcinoma patients; CTCs in breast cancer are correlated to bone marrow micrometastases	87	[55, 121]
Ficoll	density gradient centrifugation	numerous cytospins had to be evaluated because of the low sensitivity; numerous “contaminating” MNCs in the enriched cell fraction lead to false-positive results	detection of CTCs is of prognostic relevance in BCBM patients	84	[55, 122]
AccuCyte®	density-based cell separation; allows virtually complete harvesting of the red blood cells without cell lysis or wash steps	cellular damage; viable cells recovery rate	the median CTC count was 5 circulating prostate cancer cells/7.5 mL (range, 0-20)	90-91	[69, 123]
RosetteSep	unneeded cells are cross-linked with RBCs by specific antibodies to form a dense immune rose structure; unlabeled and highly purified target cells are left at the interface between plasma and density gradient centrifuge during density gradient centrifugation	cause inherent cell loss and morphologic changes during the spinning and wash steps	CTCs were detected in 54% (15/28) of MBC patients, 64% (16/25) of advanced stage HNC patients	36 ± 18	[9, 70, 95]
SPPCN	based on the surface charge of cancer cells serum protein-coated electrically charged nanoparticles can trap different cancer cells	repeated magnetic separation and washing cause cells loss	2-8 CTCs has been isolated from 1 mL of blood; only 0-1 CTC was detected from 10 healthy donors’ blood samples	50-89	[75]
DEP-FFF Device	DEP crossover frequencies of CTCs that are distinct from those of peripheral blood cell subpopulations and would permit them to be isolated from blood.	throughput limitation; Cannot be routinely applied in the biomedical and basic science labs	offer higher discrimination and throughput than earlier DEP trapping methods; not test in cancer patients	92	[7]
ApoStream®	using dielectrophoretic technology in a microfluidic flow chamber; overcomes throughput limitations; high precision and linearity of recovery of viable; cancer cells	may cause cellular damage	be used to detect FRα(+) CTCs and may have clinical utility for assessing FRα levels in cancer patients; detect EMT-CTCs among patients after neoadjuvant chemotherapy	75.4 ± 3.1; 71.2 ± 1.6	[72, 124, 125]
**Functional Assays**					
ELISPOT	enriches cells via a depletion of the CD45+ hematopoietic cells and detects proteins shed/ secreted/ released from single epithelial cancer cells; a multi-parameter analysis revealing a CTC/DTC protein fingerprint	requires efficient antigen binding and specific epitope presentation; high antigen levels demand; transition into in vitro cultures decrease cell viability and reduce detection rates	measure the release of cytokeratin-19 (CK19) and mucin-1 (MUC1) in BC; measure the release of PSA in prostate cancer;	–	[78]
CAM assay	based on CTC invasiveness compared to other cells; effective enrichment and identification based on CTC invasiveness; downstream analysis is possible.	isolation step requires more than 12 hours; biomarker dependent	capture invasive CTCs in mCRPC, mNSCLC and mPDAC	54 ± 9	[79]
Nanoroughened Surfaces	utilize the differential adhesion preference of cancer cells to nanorough surfaces	adhesion strength of cancer cells might be affected by nanotopographic sensing; may cause cellular damage	efficiently capture different kinds of cancer cells (MCF-7, MDA-MB-231, Hela, PC3, SUM-149); not test in cancer patients	> 80	[79]
TelomeScan	Detects elevated telomerase activity via a telomerase-specific replication selective adenovirus	May also detect hematopoietic stem cells for false-positive results	The sensitivity of CTC detection was 69.1% in NSCLC patients; Patients with positive EMT-CTCs at baseline had poor response to chemotherapy and decreased PFS	97	[81, 82, 126]
**Microdevices**					
eLoaD microfluidic platform	Anti-EpCAM was immobilized on gold electrodes; quantifies CTCs by using label-free electrochemical impedance;	CTCs expressing low levels of EpCAM are unlikely to be captured	perform five different assays in parallel with linear dynamic range between 16,400 and (2.6 ± 0.0003) × 10^6^ cancer cells/mL of blood; not test in cancer patients	87	[98]
NanoVelcro	utilize an anti-EpCAM-coated SiNS to achieve significantly enhanced capture of CTCs Thermoresponsive NanoVelcro chips have demonstrated the capture and release of CTCs at 37 and 4 °C	Only EpCAM-positive CTCs are detected	clinical applications of each generation for various types of solid cancers (prostate cancer, pancreatic cancer, lung cancer, and melanoma)	> 85	[99]
iMECH	deformation-based separation of CTCs from whole blood; enable label-free biomechanical profiling of individual cells for distinction; provide a low-cost yet high-throughput for single-cell level metastatic detection	detect non-metastatic cells for false-positive results; may cause cellular damage	MDA-MB-231 and MDA-MB-468 cells exhibit a loss of resistance; not test in cancer patients	95 identified as metastatic	[100]
Size-Selective Microcavity Array	separate cancer cells from the blood in accordance with differences in the size and deformability; approximately 98% of viable recovered cells; fast samples processing speed (200-1000 μL/min)	clogging of cavities; size-heterogeneity	detect approximately 97% of NCI-H358 cells in 1 mL whole blood spiked with 10-100 lung cancer cells; not test in cancer patients	> 80	[127]
PDMS microfiltration chip	PDMS microfiltration membrane; size-based separation of CTCs from whole blood	size-heterogeneity; balance the recovery rate and purity	achieved great recovery from lung cancer cells spiked blood samples; a high processing throughput of 10 mL/h; not test in cancer patients	> 90	[53]

### CTCs detachment

How to release CTCs nondestructively after catching them from the surface effectively remains a challenging problem that needs to be solved. Detachment from filters, immunoaffinity chips and other substrates using excessive stress may reduce the cell viability and potentially induce phenotypic change, resulting in the loss of valuable information regarding the isolated cells [128]. The current technologies that hold great potential for CTC detachment include enzymatic digestion, oligonucleotide-mediated aptamer release, and stimuli-responsive polymers [56–58].

Although enzymatic digestion is applied to digest the extracellular matrix and detach cells, which may reduce other cell membrane proteins and damage cell-to-cell junctions, it is still the standard method of CTC release. In recent years, many new enzymatic degradations, including alginate lyase and endonuclease, have been developed to ameliorate the cell viability and reduce cell damage [56, 129]. Aptamers are burgeoning and powerful tools used to study CTCs that provide high stability resistance to a spectrum of harsh conditions, thereby offering a noninvasive and efficient detachment technique. In addition, aptamers can be developed against binding targets in the range between small compounds and large cell membranes or transmembrane proteins on CTCs [57, 130]. Similarly, polymers can reversibly change their conformation via deformation or dissolution in response to changes in external conditions [131]. Temperature-responsive polymers have been used to control cell adhesion with the aim of recovering cells for additional analyses. The third-generation NanoVelcro chips have demonstrated the capture of CTCs at 37 °C and release at 4 °C. The temperature-dependent conformational changes of polymer brushes can alter the accessibility of the capture agent effectively with desired CTC viability and molecular integrity [100]. A dual-mode gelatin-based nanostructured coating that can achieve temperature-responsive release of CTCs from peripheral blood was presented. The cell viability was 88.3%, and the recovery rate was 93.2% [132]. Another type of polymer commonly used are pH-responsive polymers which are synthesized by linking structures with weakly acidic and basic functional groups to a hydrophobic base. They are specifically triggered by the pH of the environment (by either accepting or releasing protons), which undergoes changes in physicochemical properties [58]. The ionization of polymers can directly affect their affinity to ECM proteins because these proteins are negatively charged under physiological conditions, resulting in high cell viability and recovery [80].

### Clinical relevance of CTC

Liquid biopsy, as a noninvasive detection method, can be extracted from peripheral blood to detect the biological characteristics and molecular typing of primary tumor cells (Table 2).

**Table 2:** Clinical applications of CTCs in recent three years (from 2020 to 2022)

Cancer Type	CTCs Utility	Detection Methods	Molecular Characteristic	Main Findings/Purposes	Trial Identifier	Reference
breast cancer	prognostic value	CellSearch® system	PI3KCA, ESR1	the detection of 5 cells/7.5 mL of blood is the best cutoff point to stratify the patients’ prognosis	NA	[133]
	prognostic value; recurrence monitoring	an epithelial cell adhesion molecule–based, positive-selection microfluidic device	NA	the presence of circulating tumor DNA and circulating tumor cells after NAC in patients with early-stage TNBC was associated with significantly inferior distant DFS, DFS, and OS	NCT02101385	[134]
	therapeutic monitoring	CellSearch® system	NA	a 50% reduction in baseline apoptotic CTC count represents the optimal cut-off to differentiate between therapy response and disease progression	NA	[135]
	prognostic value	CellSearch® system	HER2, CK	CTC heterogeneity in the blood of patients is inversely associated with OS.	NA	[136]
	prognostic value; guiding therapy	CellSearch® system	HER2	first-line HER2-targeted therapy of mBC seems to reduce CTC levels greater than endocrine or chemotherapy; anti-HER2 therapy seems to be associated with lower overall CTC levels.	NA	[44]
	guiding therapy	CellSearch® system	HER2	HER2 + CTCs ≥2 associated with shorter survival and higher risk for disease progression (HR 2.16)	NA	[137]
	guiding therapy	CellSearch® system	PD-L1	CTC and platelet PD-L1 expression could predict which patients should receive immune checkpoint inhibition and as a pharmacodynamics biomarker during treatment	NA	[138]
	prognostic value	CellSearch® system	NA	mortality is on the number of CTC/7.5 mL WB in patients with mBC starting first-line chemotherapy	NCT00382018	[139]
	prognostic value	density-based isolation	TWIST1, CD24, CD44, and ALDH1	TWIST1 in EpCAM+ cells had a significant lower DFS and OS	NA	[140]
	prognostic value; therapeutic monitoring	CellSearch® system	NA	the addition of bevacizumab was associated with a PFS benefit regardless of CTC count, but an OS benefit was only observed in CTC-positive patients	NCT00601900	[141]
	prognostic value	GILUPI CellCollector	NA	evaluate the predictive value of CTC in NAC among locally advanced breast cancer patients.	NCT03732339	No Results Posted
lung cancer	diagnostic value	ISET®	NA	the ISET Rarecells test used in this study had too low a sensitivity to be used as a reliable lung cancer screening tool for patients at high-risk	NCT02500693	[142]
	prognostic value	EpCAM-independent	NA	the high number of CTC predicted adverse prognosis	NA	[143]
	prognostic value	CytoploRare Kit	NA	preoperative CTC concentration is an independent and sensitive biomarker of prognosis in patients with NSCLC	NA	[144]
	prognostic value	Microsieve membrane filter device	NA	evaluates the use of ctDNA and CTCs in predicting disease activity and drug response in lung cancer patients	NCT04254497	No Results Posted
	guiding therapy; therapeutic monitoring	ISET®	ALK	detection by FISH analysis and prevalence of escaping mutations in circulating tumor cells for the non-invasive management of lung cancer patients	NCT02372448	No Results Posted
gastric cancer	prognostic value; guiding therapy	CellSearch® system	HER2	HER2-expression on CTCs was an independent prognostic factor for both OS and PFS; the potential clinical utility of trastuzumab combined chemotherapy in patients with HER2-positive CTCs even if they are histologically HER2-negative	NA	[145]
	prognostic value	Ficoll	FGFR2	patients with FGFR2-positive CTCs (≥5 cells/10 mL blood) had significantly worse RFS	NA	[146]
	prognostic value	Ficoll	CEA	the number of EpCAM - /CEA + cells was higher in patients with stage II–IV than in patients with stage I; a lower number of CTCs indicated a higher 3-year RFS.	NA	[147]
	therapy monitoring	CTCBIOPSY	NA	compare both short-term and long-term treatment effect of laparoscopic vs. open approach on progressive gastric and rectal cancer, based on circulating tumor cell (CTC) test results and DFS	NCT02955173	No Results Posted
colorectal cancer	prognostic value	CellSearch® system	NA	elevated bCTCs and RASmut were associated with clinicopathologic features known to be associated with poor prognosis	NCT01640405NCT01640444	[148]
	prognostic value	Density gradient isolation	CEACAM5	using CEACAM5 as a dynamic poor prognostic CTC biomarker in patients with mCRC; MSI-High was identified as an unfavorable prognostic factor for tumors in patients with mCRC	NA	[149]
	therapy monitoring	EPISPOT	NA	a prospective study of a cohort of patients with metastatic colorectal cancer was conducted to demonstrate the predictive value of CTC counts for treatment response.	NCT01596790	No Results Posted
pancreatic cancer	guiding therapy; prognostic value	CellSearch® system	NA	patients with positive CTC (≥1) preoperatively had a poor prognosis despite successful tumor resection, a finding with high specificity.	NA	[150]
hepatocellular Carcinoma	prognostic value	CellSearch® system	NA	CTC count ≥3 was associated with a higher risk of postoperative extrahepatic metastases;	NA	[151]
	prognostic value	CanPatrol	Nanog	the numbers of EpCAM mRNA+ CTCs and Nanog mRNA+ CTCs were strongly correlated with postoperative HCC recurrence	NA	[152]
	prognostic value	CellSearch® system	NA	elucidate the association between the levels of CTC/CTC clusters and patients’ disease during the perioperative period; explore the molecular basis of CTC production in hepatocellular carcinoma.	NCT05297955	No Results Posted
renal cell carcinoma	prognostic value	CellSearch® system	NA	the presence of ≥3 CTCs at baseline is associated with a significantly shorter PFS and OS in patients with mRCC	NA	[153]
	prognostic value	CanPatrol-ITMCTCs	NA	no differences in the OS and DFS between the different numbers of CTCs	NA	[154]
prostate cancer	guiding therapy	Streck tubes	AR-V7	patients with detectable nuclear-localized AR-V7 in CTCs had superior survival with taxanes over ARSIs	NA	[155]
	prognostic value	VERSA	NA	a transcriptional profile detectable in CTCs can serve as an independent prognostic marker beyond AR-V7 in patients with mPC CTC can be used to identify the emergence of multiple ARSI resistance mechanisms.	NCT01942837 NCT02025010	[156]
	prognostic value	AdnaTest®	AR; AR-V7	detection of AR-V7 in CTCs is independently associated with shorter PFS and OS with abiraterone or enzalutamide; men with AR-V7-positive disease experience clinical benefits from taxane chemotherapy	NA	[157]
	prognostic value	Epic Sciences	AR	chromosomal instability of CTCs was associated with poor OS in patients treated with AR signaling inhibitors and taxanes.	NA	[158]
	prognostic value	CellSearch® system	NA	low CTC detection rate in patients with locally advanced high-risk prostate cancer; the conversion of CTCs was significantly associated with stages T3 (*P* = 0.044) and N1 (*P* = 0.002); detection of CTCs was not significantly associated with overall survival (*P* > 0.40)	NCT01800058	[159]
bladder Cancer	prognostic value	Telomerase-based technique	NA	detect tCTC levels in bladder cancer patients in different cohorts; clarify how tCTC levels vary with the natural history of bladder cancer; observe whether tCTCs provide new information.	NCT02246738	No Results Posted
gestational choriocarcinoma	guiding therapy	NanoVelcro system	NA	patients with ≥4 CTCs were more likely to develop chemoresistance than those with < 4 CTCs (*P* < 0.001)	NA	[127]
gynaecological malignancy	prognostic value	CellSearch® system	NA	patients with ≥ 1 CTC at baseline had significantly shorter OS and PFS than CTC-negative patients	NA	[160]

### Studies based on CTC count

Higher CTC counts in patients’ peripheral blood have been reported to be associated with a poor prognosis in various types of cancers, including colorectal cancer, breast cancer, lung cancer，pancreatic cancer and so on [16, 79, 160–162]. It has been proven that the presence of ≥3 CTCs per 7.5 mL of peripheral blood is a strong predictor of progression-free survival (PFS) reduction, whereas the detection of < 3 CTCs per 7.5 mL indicates better overall survival (OS) [133, 163]. Initial CTC counts as well as early changes after treatment initiation are closely related to the primary tumor size, the number of metastases, and the PFS reduction in patients with breast cancer [27, 164, 165]. CTC counts increase with tumor progression and development of distant metastases [166]. It has been reported that the area under receiver operating characteristic (ROC) curve for CTC count in forecast of distant metastasis was 0.783 [167].

CTC detection is a potential novel approach to assess the efficacy of neoadjuvant chemotherapy (NAC) [168]. Indeed, the results of studies published in the past 5 years, involving thousands of patients with breast cancer, have demonstrated that the CTC counts before and after neoadjuvant therapy are predictive of the risk of disease relapse [134]. Patients with ≥4 CTCs were more likely to be resistant to chemotherapy than those with < 4 CTCs, indicating that the CTC count is a promising indicator in the evaluation of biological activities and the chemotherapy response in gastric carcinoma (GC) patients [127]. CTCs may be a practical surrogate marker with the chemotherapy response since chemotherapy leads to a rapid decline in CTC counts with a 50% reduction in baseline apoptotic CTC count [135, 160].

Data obtained in animal models indicate that blood dissemination of cancer cells occurs early during tumor development, which may provide the possibility to explore CTCs as marker for early detection [169]. It has been demonstrated that CTC-positive chronic obstructive pulmonary disorder (COPD) patients were examined with lung nodules 1 to 4 years after CTC detection, leading to prompt surgical resection and histopathological type of early-stage lung cancer. Follow-up studies conducted one-year post-surgery showed no tumor recurrence [170]. It seemed that CTC as a sentinel of tumor development could save patient lives – especially in asymptomatic cancers for which no routine screening methods are available. The initial encouraging results of the pilot study in patients with COPD generated public attention, but the results of the later validation cohort study confirmed that CTC detection is not suitable for lung cancer early detection [142]. The low sensitivity of CTCs for early cancer detection might be explained as the gradient difference of tumor cells counts between the tumor-draining vessels and the peripheral veins [171, 172]. Metastases present in lymph nodes or distant organs promote the pool of CTCs in peripheral blood in later tumor stages, which considerably increases CTC counts. In conclusion, CTC plays a significant role in early detection, dynamic monitoring, efficacy evaluation and prognosis judgment.

### Studies based on molecular characteristics of CTC

In addition to pure quantitative analyses, the use of CTCs as a tumor surrogate was concerned as one of the main concepts studied in clinical trials. CTCs from patient peripheral blood may be a novel and attractive noninvasive alternative for assessing tumor heterogeneity, molecular tumor characteristics and changes during treatment.

Many studies have identified genes that can be used as prognostic markers by CTC detection, including HER2, ESR1, PI3KCA, PSMA, MYC, TP53 and so on [173–176]. Several studies have demonstrated the feasibility of evaluating HER2 status of CTCs in BC using CellSearch® [136, 177, 178]. Jaeger and colleagues have found unusual inconsistency of HER2 expression between CTCs and the primary tumor in early breast cancer. They have detected HER2-positive CTCs in peripheral blood from patients with HER2-negative breast cancer [178]. Current studies have reported that HER2-negative breast cancer patients with HER2-expressing CTCs can still benefit from trastuzumab therapy [137]. ESR1 gene mutations have reported as a biomarker for resistance to endocrine therapy in BC. ESR1 mutations which rarely detected at the beginning of first-line endocrine therapy were significantly enriched during disease progression, suggesting that ESR1 mutations conferred endocrine resistance in metastatic breast cancer [179]. Mastoraki et al. investigated epigenetic silencing of ESR1 and its effect on endocrine therapy response. ESR1 methylation was observed in 27.8% (10/36) of CTC-positive samples and was associated with non-response to treatment in peripheral blood samples from everolimus/exemestane-treated patients [180]. Changes in CTC count based on PSMA status were determining by EPIC Sciences technology in a phase 2 trial evaluated the efficacy and safety of BIND-014 in prostate cancer patients. Interestingly, PSMA-positive CTCs were reduced preferentially compared with the baseline, indicating the effect of PSMA-positive CTCs as biomarkers and monitors for PSMA-based treatment [181]. Gene expression profiling of CTCs in metastatic breast cancer suggested that CTCs associated with brain metastasis had increased activity of the Notch signaling pathways [182]. Another study revealed that overexpression of MYC and copy-number gain of SEMA4D (a mediator of blood–brain barrier transmigration) were novel markers for brain metastasis through a genome-wide assessment of CTC lines established from breast cancer patients [183].

The appearance of inhibitors such as PD-1 or PD-L1 has demonstrated interesting results in certain metastatic cancers. In NSCLC, CTC status was assessed with CellSearch® and PD-L1 staining methods at baseline, and at 3 and 6 months in patients treated with nivolumab. Patients with PD-L1 negative CTCs at 6 months gained a clinical benefit, while patients with PD-L1 positive CTCs experienced tumor progression [184]. A recent study using CellSearch® to continuously collect blood, utilized PD-L1 antibodies to measure CTCs and platelets in both patients with metastatic breast cancer and healthy subjects. More than 40% patients (52/124, 42%) detected ≥5 CTCS / 7.5 mL whole blood, and 21 (40%) were PD-L1 positive for CTCs [138]. These studies showed that PD-L1 expression existed independently on CTCs and could play a role as a pharmacodynamic biomarker predicting which patients should receive immune checkpoint suppression and therapy.

## Conclusions and future perspectives

The novel discovery of CTCs as a liquid biopsy had a revolutionary effect on early diagnosis, metastasis detection and individualized treatment of tumors [134, 162, 163]. Despite the advantages above, the clinical use of CTCs is hindered by considerable challenges because of the heterogeneity, fragility, singularity and incomplete gene expression expertise of CTCs. Methods for isolating, detaching, and detecting these cells in blood from cancer patients have been rapidly developed to address the need for increased specificity, sensitivity, and throughput. The most commonly used detection methods are based on specific surface antigens, physical properties and functional properties of CTCs [43, 80, 185]. CTCs extracted from patient samples can be used for further studies to develop the best treatment regimen, conduct effective disease surveillance and discover new drug targets for molecular and genetic analyses [14].

However, many problems have not been solved. First, several biological questions remain, such as, what determines the tendency of CTC metastases and which pathways could be targeted for metastatic restraint of CTCs. Also, since trafficking of CTCs may be regulated by circadian rhythm, the distribution of CTCs in circulation may not be uniform. Patients with zero CTC detected at a given time point may not be CTC-free [186]. Repeated blood draws to clarify the temporal distribution of CTCs in patients are not realistic. IVFC could help monitoring CTCs dynamically to reduce the false negative rate, but it is still need more preclinical research to prove whether it can be applied in patients to solve the false negative results caused by detection time.

Secondly, although there are many CTC isolation and detection techniques, different CTC-positive ratios are reported from the usage of different methods. Therefore, it is necessary to establish and improve the standardized protocols off CTC-related detection methods as soon as possible. The Horizon 2020 SPIDA4P aims to develop and implement a comprehensive portfolio of 22 pan-European CEN and ISO standards documents, driving the standardization of preanalytical workflows applied to personalized medicine (www.spidia.eu). Due to the high senescence of CTCs, blood is usually placed in blood collection tubes with preservatives for long term preservation, which could result in the loss of viable CTC cells [187]. It is also significant to consider the volume of blood samples to unify CTC isolation and identification protocols since CTCs are rare in the bloodstream [187]. In addition, how many CTCs are required for a genic panorama of the donor is another problem under solved.

Thirdly, different CTC techniques used for detecting the same sample may obtain completely different results; thus, how to choose the most suitable CTC detection method is also a major problem that currently needs to be solved [83, 188]. A study aimed to evaluate how two different isolation techniques, evolving the physical (Parsortix®) and biological (MACS®) separation techniques, affect cell morphology. The researchers found that in the MBC patient cohort, the morphological features of CTCs were dependent on the separation process. CTCs with a preserved cell morphology were detected after physical separation while the identification of the cell morphology was difficult due to the degeneration of CTCs after biological separation [111]. A comparative study indicated that although the EpCAM-dependent CTC enrichment was superior in terms of specificity compared to label-free CTC enrichment, it is more suitable to choose size-dependent enrichment approaches in consideration of the evaluation of CTC molecular characterization [189]. Regardless, not all CTC methods are appropriate for downstream analysis, such as DNA analysis. It is also challenge to find the most suitable detection method to be applied in different tumor screening setting. Devices based on positive selection achieve the high purity in clinical applications but lose CTC subpopulations including EMTed CTCs, clusters, and CTCs cloaked by blood cells. On the contrary, negative selection-based techniques are theoretically capable of enriching all potential CTC subpopulations but with low purity. Along with the devices based on biological properties, techniques isolating and detecting CTCs based on their physical properties appear suitable for use in a clinical cytopathology laboratory for identification of CTC morphology and evaluation of CTC molecular characterization [112].

In addition, the current studies investigating the clinical application of CTCs mostly focus on advanced or metastatic cancers and rarely involve their application in early-stage cancer. Can CTCs be detected reliably in early disease and ca be used to routinely guide cancer patient care are still unanswerable problems. The number of CTCs detected in the blood of patients with early-stage cancer is lower than that in patients with metastatic disease, requiring higher sensitivity. Therefore, is it necessary to apply such a high-cost technique for the detection of rare CTCs in the patients with early-stage cancer?

Finally, whether the CTCs obtained by these CTC detection techniques are truly representative of the heterogeneity of the primary tumor or whether these techniques could detect those so-called CTCs remains an unanswered question. A recent study demonstrated that transcriptional profiles may be altered when cells leave hypoxic primary lesions and enter the well-oxygenated bloodstream [190].

CTC analysis is a simple and feasible liquid biopsy technique that has attracted great attention and achieved great success, although there are still some problems to be solved. The further development of CTC diagnostic technology should be of great value in the individualized treatment of cancers.

## Data Availability

Data sharing is not applicable to this article as no datasets were generated or analyzed during the current study.

## Abbreviations

CAM: Collagen adhesion matrix
CCL2: Chemokine C-C motif ligand 2
COPD: Chronic obstructive pulmonary disorder
CTC: Circulating tumor cell
CTL: Cytotoxic T lymphocyte
DEP: Dielectrophoresis
DEP-FFF: Dielectrophoretic field-flow fractionation
ECM: Extracellular matrix
EGFR: Endothelial growth factor receptor
ELISPOT: Epithelial Immuno-spot
EMT: Epithelial-mesenchymal transition
EpCAM: Epithelial cell adhesion molecule
HER2: Human epidermal growth factor receptor 2
IVFC: Fluorescence in vivo flow cytometry
MDSC: Myeloid-derived suppressor cell
MHC-I: Major histocompatibility complex-I
MMP: Matrix metalloproteinase
NAC: Neoadjuvant chemotherapy
NK cell: Natural killer cell
PD-L1: Programmed cell death-ligand 1
PSA: Prostate specific antigen
ROC: Receiver operating characteristic
SPPCN: Superparamagnetic positively charged nanoparticle
TGF-β: Transforming growth factor-β
VEGF: Vascular endothelial growth factor
